# Transcriptomes of Different Tissues of Flax (*Linum usitatissimum* L.) Cultivars With Diverse Characteristics

**DOI:** 10.3389/fgene.2020.565146

**Published:** 2020-11-30

**Authors:** Alexey A. Dmitriev, Roman O. Novakovskiy, Elena N. Pushkova, Tatiana A. Rozhmina, Alexander A. Zhuchenko, Nadezhda L. Bolsheva, Artemy D. Beniaminov, Vladimir A. Mitkevich, Liubov V. Povkhova, Ekaterina M. Dvorianinova, Anastasiya V. Snezhkina, Anna V. Kudryavtseva, George S. Krasnov, Nataliya V. Melnikova

**Affiliations:** ^1^Engelhardt Institute of Molecular Biology, Russian Academy of Sciences, Moscow, Russia; ^2^Federal Research Center for Bast Fiber Crops, Torzhok, Russia; ^3^All-Russian Horticultural Institute for Breeding, Agrotechnology and Nursery, Moscow, Russia; ^4^Moscow Institute of Physics and Technology, Dolgoprudny, Russia

**Keywords:** flax, *Linum usitatissimum* L., transcriptome sequencing, comparative genomics, genotype-phenotype, gene expression, DNA polymorphism

## Introduction

Flax (*Linum usitatissimum* L.) is widely used to produce fiber and seed. Linseed is the richest source of omega-3 fatty acids, which reduce the risk of cancer and cardiovascular diseases, and lignans, which have antibacterial, antifungicide, antioxidant, and anticarcinogenic activities, and also contains easily digestible proteins, dietary fibers, vitamins, and minerals (Muir and Westcott, 2003; Goyal et al., 2014; Imran et al., 2015; Kezimana et al., 2018; Parikh et al., 2018; Cullis, 2019; Mali et al., 2019). Linseed is used in environment-friendly paints and varnishes and also in animal feed to obtain products with increased content of polyunsaturated fatty acids (Kouba and Mourot, 2011; Singh et al., 2011; Goyal et al., 2014). Flax fiber is valuable for the production of textile and composite materials (Costa et al., 2018; Baley et al., 2019). To obtain high and stable yields of organic flax products, it is necessary to cultivate varieties that are resistant to adverse environmental factors and possess a complex of economically valuable traits. The use of traditional methods of breeding requires up to 12–15 years to create a new cultivar. Biotechnologies, including genome editing and marker-assisted and genomic selection, can significantly increase the accuracy and efficiency of the breeding process (Dwivedi et al., 2018; Cobb et al., 2019; Gionfriddo et al., 2019; Mascher et al., 2019; Varshney et al., 2019). For the development and introduction of biotechnologies into practice and breeding of improved cultivars of *L. usitatissimum*, large-scale studies of genomes and transcriptomes on representative sets of flax samples with diverse characteristics are needed.

The flax genome was sequenced and a significant number of transcriptomic studies were performed (Wang et al., 2012; You et al., 2018; Cullis, 2019; Akhmetshina et al., 2020) that laid the foundation for identification of genes that are responsible for valuable traits. Using transcriptome sequencing, the search was performed for genes that are associated with the following flax features:

1) Fiber characteristics in varieties Baihua (Long et al., 2012), Zhongya 2 (Guo et al., 2017), and Mogilevsky (Gorshkov et al., 2017; Mokshina et al., 2017; Gorshkova et al., 2018);
2) Seed characteristics in varieties NEW and Shuangya 4 (Xie et al., 2019);
3) Response to *Fusarium oxysporum* infection in varieties CDC Bethune, Lutea (Galindo-Gonzalez and Deyholos, 2016), Dakota, #3896, AP5, TOST (Dmitriev et al., 2017), and Nike (Preisner et al., 2018);
4) Response to drought in variety T-397 (Dash et al., 2017);
5) Response to unfavorable soil pH and content of macro- and microelements in varieties Heiya No.19 (Yu et al., 2014), Hermes, TMP1919, Lira, Orshanskiy (Dmitriev et al., 2016a), CDC Bethune, Stormont Cirrus (Dmitriev et al., 2016b), Norlin, Mogilevsky (Dmitriev et al., 2019), and Agatha (Wu et al., 2019).

However, in most studies, only one or two cultivars/lines were used, but for the comparative analysis and search for a gene function, much more genotypes with diverse agronomically important traits should be investigated. In the present work, we performed transcriptome sequencing of five different tissues of six flax cultivars/lines with a diverse feature set.

## Materials and Methods

### Plant Materials

Five flax cultivars (Alizee, Atlant, Diplomat, LM98, and Universal) and one line (#3896) were chosen for the present study based on their breeding value and differences in agronomically important parameters such as productivity, fiber and seed characteristics, and resistance to stresses. The characteristics of examined genotypes are represented in Table 1 (Ryzhov et al., 2012; Rozhmina and Loshakova, 2016; Pavlova et al., 2018; Kolotov, 2020; Rozhmina et al., 2020). Seeds were obtained from the Institute for Flax (Torzhok, Russia).

**Table 1:** Characteristics of six examined flax cultivars/lines.

**Name**	**Type**	**Height, cm**	**Productivity, c/ha**			**Fiber content, %**	**Long fiber, %**	**Long fiber number**	**Oil content, %**	**Weight of 1,000 seeds**	**Vegetation period, days**	**Resistance to lodging, score**	**Resistance, %**		
			**Straw**	**Seed**	**Fiber**								**Fusarium wilt**	**Rust**	**pH 6.2**
Alizee	Fiber flax	71	75.3	8.6	23.5	31.2	17.3	12.3	37.1	5.44	90	9.0	54.0	99.1	44.6
Atlant	Fiber flax	84	80.7	8.7	20.5	25.4	19.6	14.2	38.4	4.29	82	8.0	90.9	88.4	75.3
Diplomat	Fiber flax	69	63.2	10.7	21.1	33.4	13.4	12.9	37.5	4.65	77	8.5	80.2	99.7	31.3
Universal	Fiber flax	73	56.4	10.6	17.2	30.5	13.7	12.7	38.9	4.79	71	7.5	92.5	89.4	70.9
#3896	Linseed	66	24.3	16.0	4.7	19.4	6.8	-	44.0	7.05	91	8.5	94.4	87.9	95.2
LM98	Linseed	60	52.9	19.1	11.4	21.6	9.4	-	42.8	5.34	108	8.0	6.7	77.5	58.4

*c/ha—centners per hectare*.

Flax seeds were sterilized in 1% sodium hypochlorite for 2 min. Seedlings were grown in Petri dishes for 7 days, and then roots and shoots were collected from five plants for each cultivar/line and frozen in liquid nitrogen until further use. Also, plants were grown in the greenhouse for 6 weeks to the flowering stage, and after that, plant materials were collected from leaves, stems (the upper and middle part of the plant), and flowers of five plants for each cultivar/line and immediately frozen in liquid nitrogen until further use. Samples were stored at −75°C before RNA extraction.

### RNA Extraction and Transcriptome Sequencing

RNA was extracted from pools of five plants for each combination of tissue-genotype. Plant materials were homogenized using MagNA Lyser (Roche, Switzerland) in 600 μl of RNA lysis buffer from a Quick-RNA Miniprep Kit (Zymo Research, United States) with solid-glass beads (Sigma-Aldrich, United States) and then RNA was extracted according to the Quick-RNA Miniprep Kit protocol with in-column DNase I treatment. Quality and concentration of RNA were evaluated using 2100 Bioanalyzer (Agilent Technologies, United States) and a Qubit 2.0 fluorometer (Thermo Fisher Scientific, United States). RIN (RNA Integrity Number) values were more than 8 and close between samples. NEBNext Poly(A) mRNA Magnetic Isolation Module (New England Biolabs, United Kingdom) and NEBNext Ultra II Directional RNA Library Prep Kit for Illumina (New England Biolabs) were used for isolation of mRNA from 1 μg of total RNA and cDNA library preparation according to the manufacturer's protocols. In total, 30 libraries were obtained—from roots and shoots of seedlings and leaves, flowers, and stems of adult plants for each of six flax cultivars/lines. The quality of cDNA libraries was evaluated using 2100 Bioanalyzer (Agilent Technologies)—they had an optimal length distribution and were free of adapter dimers. After that, the libraries were sequenced on NextSeq 500 (Illumina, United States) with a read length of 86 bp.

### Preliminary Data Analysis

Transcriptome sequencing of 30 cDNA libraries from five different tissues (leaves, flowers, stems, seedling roots, and seedling shoots) of six flax cultivars/lines (#3896, Alizee, Atlant, Diplomat, LM98, and Universal) was performed, and from 6 to 16 million reads were obtained for each library. The raw data were deposited in the NCBI Sequence Read Archive (SRA) under the BioProject accession number PRJNA634481.

Reads for each library were trimmed with Trimmomatic (Bolger et al., 2014) and mapped to the NCBI representative *L. usitatissimum* genome (GenBank assembly: GCA_000224295.2) using STAR (Dobin et al., 2013), and plots were generated using MultiQC (Ewels et al., 2016). For the majority of samples, more than 87% of reads were uniquely mapped to the *L. usitatissimum* genome and about 8–10% of reads were mapped to several loci (Supplementary Data 1).

For evaluation of gene expression in examined flax tissues and genotypes, reads mapped to the *L. usitatissimum* genome were quantified using BEDTools (Quinlan and Hall, 2010). Expression levels were quantified as read counts per million (CPM) for 200-bp intervals (in case of absence of reads aligned to the particular region, intervals were increased). Obtained results are represented in Supplementary Data 2. This table is a valuable resource for differential expression analysis.

**Figure 1:**
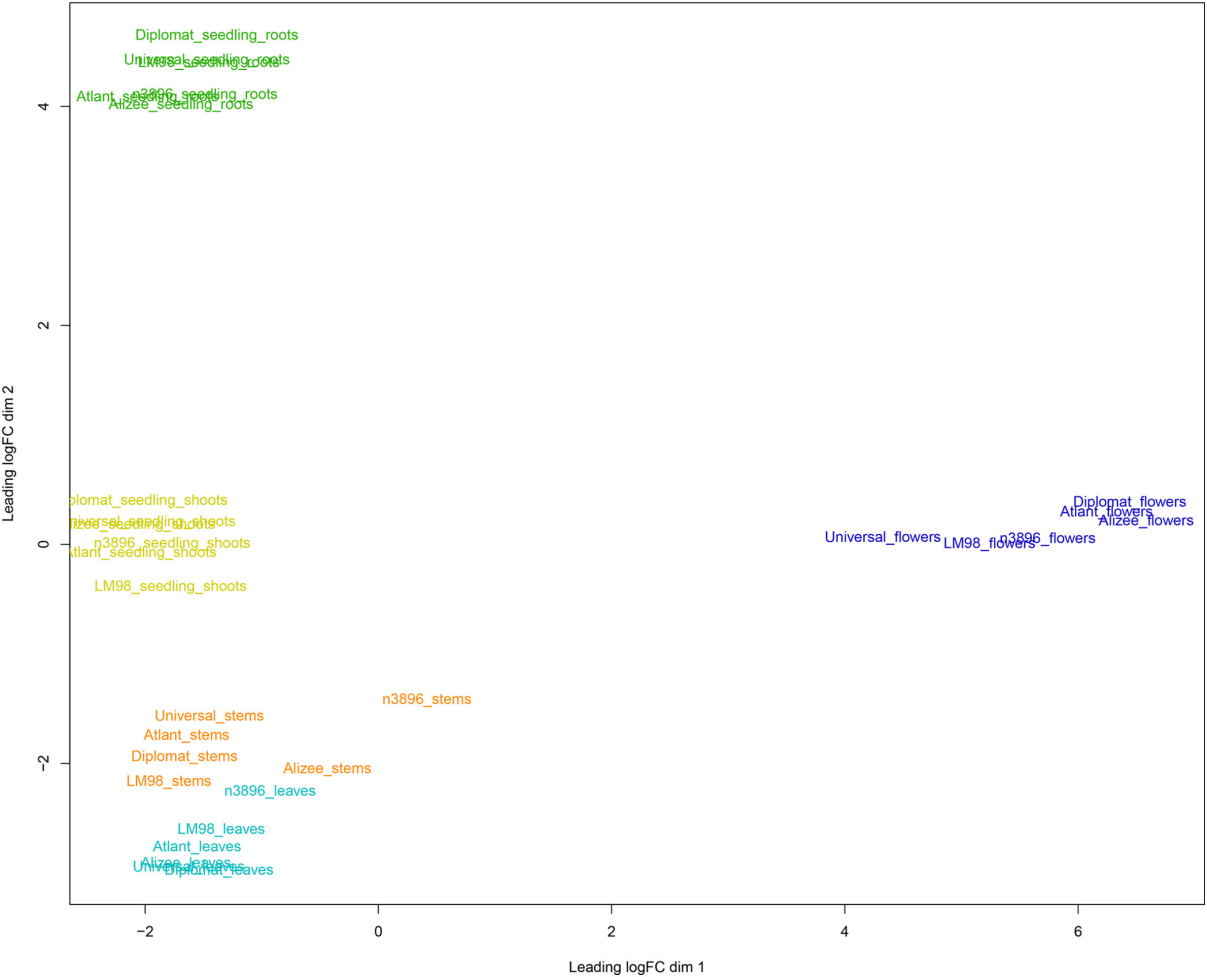
Multidimensional scaling plot for gene expression profiles of five tissues (leaves, flowers, stems, seedling roots, and seedling shoots) of six flax cultivars/lines (#3896, Alizee, Atlant, Diplomat, LM98, and Universal). Different tissues are marked with different colors: leaves—cyan, flowers—blue, stems—orange, seedling roots—green, and seedling shoots—lime.

For visualization of differences between gene expression profiles of flax tissues and genotypes, multidimensional scaling (MDS) plots were generated using edgeR (Robinson et al., 2010). MDS for 30 flax samples (five tissues of six genotypes) from the current project are represented in Figure 1. As can be seen from the figure, samples were grouped according to the type of plant material: flowers and roots formed two distant groups, while leaves, stems, and seedling shoots were close to each other. Next, the present data were combined with the data from four NCBI BioProjects, in which transcriptome sequencing was performed for flax shoots (PRJNA229810), bast fiber and xylem (PRJNA251268), roots (PRJNA412801), and developing seeds (PRJNA539945). We used only forward reads and trimmed them to 70 nucleotides to unify data and reduce the batch effect. Grouping of expression data for five different research projects, including the current one, was again consistent with the type of plant material. Three groups were revealed: group 1—flowers and seeds, group 2—roots, and group 3—leaves, stems, and shoots (Supplementary Data 3). This points to the quality of the obtained data and the possibility of a joint analysis of expression data from several research projects that is important for the identification of common regularities in gene expression for particular flax organs and tissues.

Due to the sequencing of a representative set of tissues and genotypes (five tissues of six cultivars/lines), the obtained data are the basis for gene expression analysis in a particular tissue that is important for understanding the key molecular processes occurring in flax plants. Moreover, using these data, the search for genes with the most significant differences in expression between flax genotypes with diverse characteristics can be performed that is necessary for revealing associations between cultivar/line phenotype and gene expression profile. For example, we compared gene expression levels between groups of two linseed and four fiber flax genotypes under study. As annotation is currently absent for the NCBI representative flax genome or other flax genome assemblies (https://www.ncbi.nlm.nih.gov/genome/browse/#!/eukaryotes/6953/), the representative genome (GenBank assembly: GCA_000224295.2) was divided into 1,000-bp intervals, the expression level was quantified as CPM for each interval, and differential expression analysis was performed using edgeR (Robinson et al., 2010). For each tissue, genomic regions were sorted according to the score calculated as –log(*p-*value)^*^abs(log*FC*), where p-value was estimated using quasi-likelihood methods (Lund et al., 2012) and *FC* (fold change) was equal to the ratio of average CPM in the fiber flax group to average CPM in the linseed group. The highest number of differentially expressed transcripts between linseed and fiber flax genotypes was revealed for seedling shoots and leaves, while the lowest was revealed for flowers (Supplementary Data 4–8).

Our data are also valuable for preliminary analysis of the expression of particular genes, gene families, or genes involved in the same pathway. This can be performed using Supplementary Data 1—knowing the coordinates of particular genes in the flax genome, one can find data on their expression in different genotypes and tissues. An example of such analysis is presented in our previous work on expression of cinnamyl-alcohol dehydrogenase (CAD) encoding genes in roots of flax cultivars/lines susceptible (TOST and AP5) and resistant (#3896 and Dakota) to *F. oxysporum* infection under control and the biotic stress conditions (Novakovskiy et al., 2019). Basing on the data of the present study, we performed a similar analysis of expression of 13 *CAD* genes in five tissues of six examined cultivars/lines. The results are represented in Supplementary Data 9. Tissue-specific expression was identified—*CAD2A, CAD4B, CAD5A, CAD5B*, and *CAD6* genes were expressed predominantly in seedling roots, while *CAD3B* was expressed in seedling shoots. Genotype-specific expression profiles were also observed, especially for *CAD2B, CAD3A, CAD4A, CAD7*, and *CAD8* genes. It is worth noting that the present data on expression profiles of *CAD* genes in seedling roots are highly concordant with the results of our aforementioned work (Novakovskiy et al., 2019). In both studies, *CAD6* had the highest expression within *CAD* genes in roots free from *F. oxysporum* infection, and *CAD3A* had the lowest one; expression levels of the other genes were also very similar between studies, indicating the reproducibility of our experiments.

Our dataset can also be used to search for polymorphisms in expressed regions of the genome within the studied flax genotypes. As an example, variant calling was performed using VarScan (Koboldt et al., 2012) for 13 *CAD* genes, and the largest number (eight) of single-nucleotide polymorphisms (SNPs) was identified for *CAD6*—positions CP027622.1 2160315, CP027622.1 2161357, CP027622.1 2161475, CP027622.1 2161540, CP027622.1 2162146, CP027622.1 2162234, CP027622.1 2162246, and CP027622.1 2162410 according to the NCBI representative *L. usitatissimum* genome GCA_000224295.2. Therefore, this gene may be of interest for the DNA certification of flax cultivars.

The present dataset is especially valuable for revealing trends of interest at the level of gene expression or DNA polymorphisms in expressed genomic regions. However, the validation of the identified trends on extended sample sets is necessary, and for these purposes, other approaches, such as quantitative PCR and targeted sequencing, are more appropriate. Besides, our data are valuable for obtaining complete flax genome annotation, whose absence for the NCBI representative *L. usitatissimum* genome complicates molecular genetic studies of this crop.

## Conclusions

The obtained data on 30 flax transcriptomes are the basis for the evaluation of expression of genes of interest in particular tissues and genotypes, search for genes with differential expression between genotypes with diverse characteristics, identification of polymorphisms in particular genes, and assessment of genetic diversity. Such information is necessary to establish associations between gene expression or DNA polymorphisms and valuable traits. Thus, the present dataset opens up novel opportunities for functional research, development of genome editing, and marker-assisted and genomic breeding. It creates the necessary basis for the effective application of biotechnology approaches on flax that will allow the breeding of cultivars with desirable characteristics.

## Data Availability Statement

The datasets generated for this study can be found in online repositories. The names of the repository/repositories and accession number(s) can be found at: https://www.ncbi.nlm.nih.gov/sra/~PRJNA634481.

## Author Contributions

AD, TR, and NM conceived and designed the work. RN, EP, TR, NB, AB, VM, LP, ED, and AS performed the experiments. AD, TR, AZ, AK, GK, and NM analyzed the data. AD, EP, GK, and NM wrote the manuscript. All authors read and approved the final manuscript.

## Conflict of Interest

The authors declare that the research was conducted in the absence of any commercial or financial relationships that could be construed as a potential conflict of interest.
