# Supplementary material for: Transcriptomes of Different Tissues of Flax (Linum usitatissimum L.) Cultivars With Diverse Characteristics
Source: Front Genet. 2020 Nov 30;11:565146. doi: 10.3389/fgene.2020.565146 (PMC7755106; doi:10.3389/fgene.2020.565146)
Supplement: Supplementary file 1 [file Data_Sheet_1.pdf]

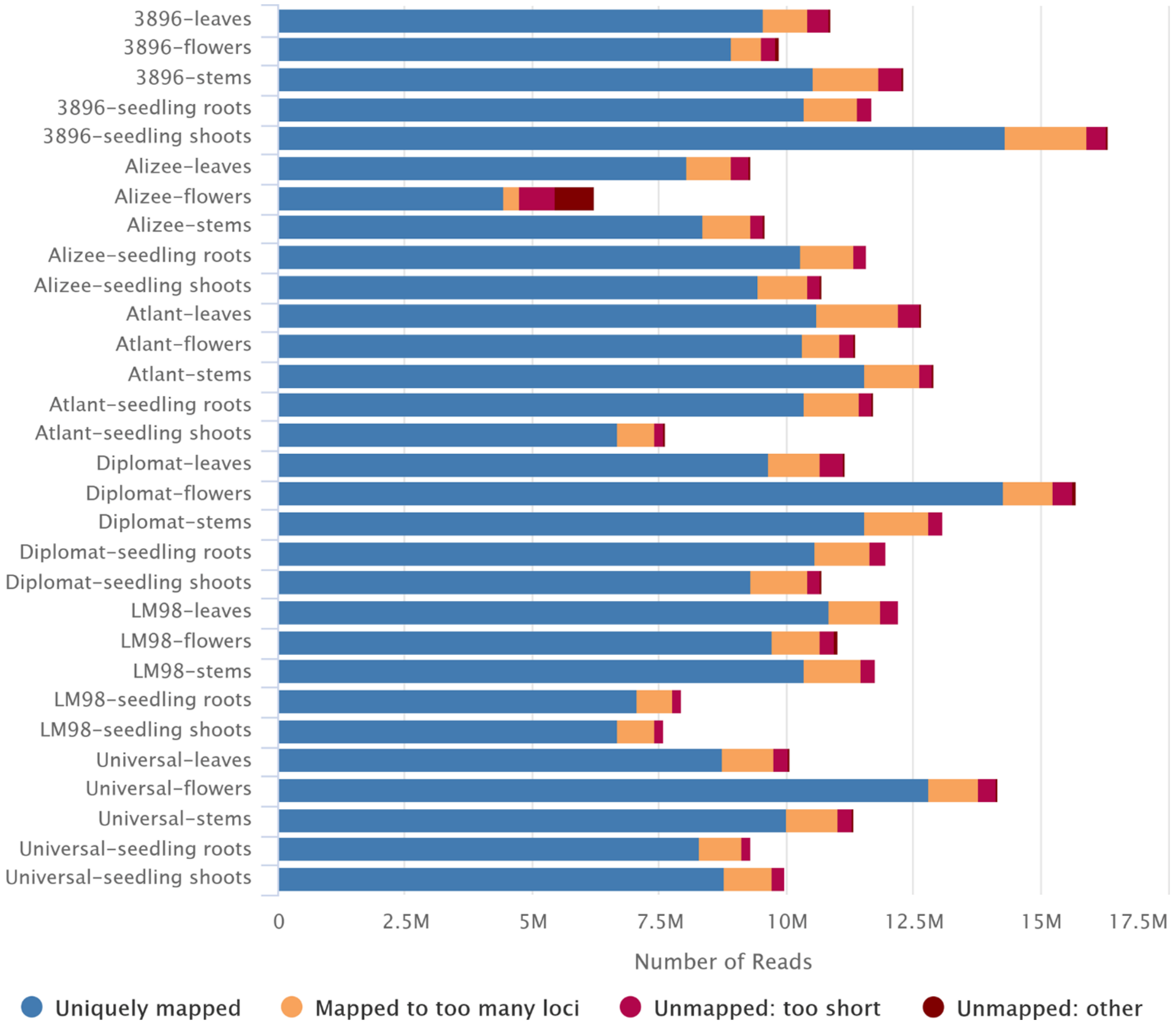

Supplementary Data 1. Statistics of transcriptome sequencing data for leaves, flowers, stems, seedling roots, and seedling shoots of six flax cultivars/lines: #3896, Alizee, Atlant, Diplomat, LM98, and Universal. Reads were mapped to the NCBI representative *L. usitatissimum* genome (GenBank assembly: GCA_000224295.2) using STAR and plots were generated by MultiQC.
