# Supplementary material for: Transcriptomes of Different Tissues of Flax (Linum usitatissimum L.) Cultivars With Diverse Characteristics
Source: Front Genet. 2020 Nov 30;11:565146. doi: 10.3389/fgene.2020.565146 (PMC7755106; doi:10.3389/fgene.2020.565146)
Supplement: Supplementary file 3 [file Data_Sheet_3.pdf]

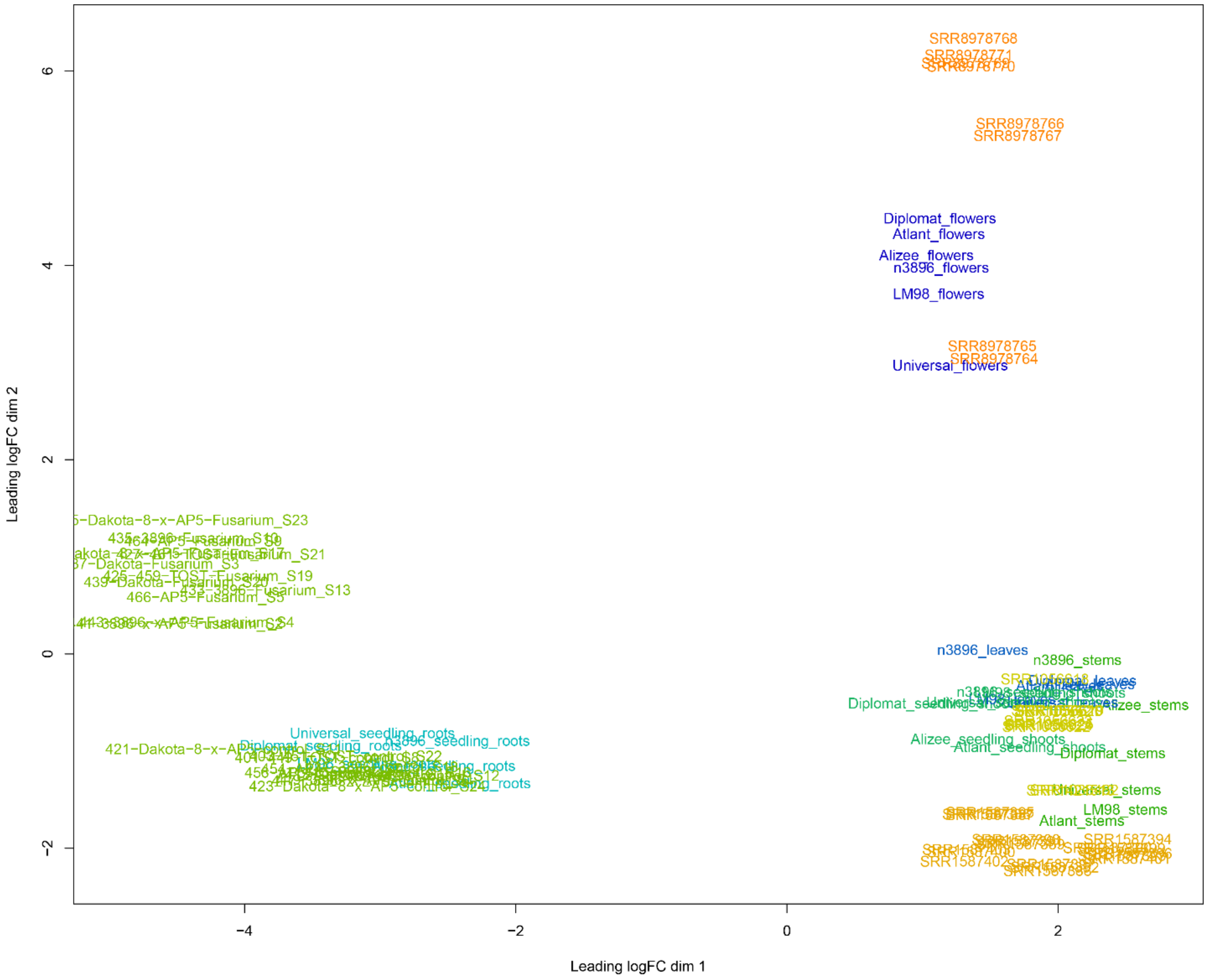

Supplementary Data 3. Multidimensional scaling plot for gene expression profiles of 5 tissues (leaves, flowers, stems, seedling roots, and seedling shoots) of 6 flax cultivars/lines (#3896, Alizee, Atlant, Diplomat, LM98, and Universal) from the current project combined with the data from four NCBI BioProjects, in which transcriptome sequencing was performed for flax shoots (PRJNA229810), bast fiber and xylem (PRJNA251268), roots (PRJNA412801), and developing seeds (PRJNA539945). Different tissues from the current project, as well as four NCBI BioProjects, are marked with different colors. Current project: leaves – blue, flowers – dark blue, stems – green, seedling roots – cyan, seedling shoots – dark green; NCBI BioProjects: shoots (PRJNA229810) – lime, bast fiber and xylem (PRJNA251268) – light orange, roots (PRJNA412801) – light green, seeds (PRJNA539945) – orange.

Samples used for the multidimensional scaling plot

BioProject	Tissue	Samples
**Current project (PRJNA634481)**	leaves	n3896_leaves<br>Alizee_leaves<br>Atlant_leaves<br>Diplomat_leaves<br>LM98_leaves<br>Universal_leaves

	flowers	n3896_flowers<br>Alizee_flowers<br>Atlant_flowers<br>Diplomat_flowers<br>LM98_flowers<br>Universal_flowers
	stems	n3896_stems<br>Alizee_stems<br>Atlant_stems<br>Diplomat_stems<br>LM98_stems<br>Universal_stems
	seedling roots	n3896_seedling_roots<br>Alizee_seedling_roots<br>Atlant_seedling_roots<br>Diplomat_seedling_roots<br>LM98_seedling_roots<br>Universal_seedling_roots
	seedling shoots	n3896_seedling_shoots<br>Alizee_seedling_shoots<br>Atlant_seedling_shoots<br>Diplomat_seedling_shoots<br>LM98_seedling_shoots<br>Universal_seedling_shoots
**PRJNA229810**	shoots	SRR1038482<br>SRR1056618<br>SRR1056619<br>SRR1056620<br>SRR1056621<br>SRR1056622<br>SRR1056623<br>SRR1056624<br>SRR1056625<br>SRR1421513
**PRJNA251268**	bast fiber and xylem	SRR1587386<br>SRR1587387<br>SRR1587388<br>SRR1587389<br>SRR1587390<br>SRR1587391<br>SRR1587392<br>SRR1587393<br>SRR1587394<br>SRR1587395<br>SRR1587396<br>SRR1587397<br>SRR1587398<br>SRR1587399<br>SRR1587400<br>SRR1587401<br>SRR1587402<br>SRR1587403

**PRJNA412801**	roots (control)	401-449-TOST-control_S8<br>403-451-TOST-control_S22<br>409-3896-control_S12<br>411-3896-control_S18<br>413-Dakota-control_S15<br>415-Dakota-control_S16<br>417-3896-x-AP5-control_S6<br>419-3896-x-AP5-control_S1<br>421-Dakota-8-x-AP5-control_S11<br>423-Dakota-8-x-AP5-control_S24<br>454-AP5-control_S7<br>456-AP5-control_S14
	roots (infected with *Fusarium oxysporum* f. sp. *lini*)	425-459-TOST-Fusarium_S19<br>427-461-TOST-Fusarium_S21<br>433-3896-Fusarium_S13<br>435-3896-Fusarium_S10<br>437-Dakota-Fusarium_S3<br>439-Dakota-Fusarium_S20<br>441-3896-x-AP5-Fusarium_S2<br>443-3896-x-AP5-Fusarium_S4<br>445-Dakota-8-x-AP5-Fusarium_S23<br>447-Dakota-8-x-AP5-Fusarium_S17<br>464-AP5-Fusarium_S9<br>466-AP5-Fusarium_S5
**PRJNA539945**	seeds	SRR8978764<br>SRR8978765<br>SRR8978766<br>SRR8978767<br>SRR8978768<br>SRR8978769<br>SRR8978770<br>SRR8978771
