# Supplementary material for: Transcriptomes of Different Tissues of Flax (Linum usitatissimum L.) Cultivars With Diverse Characteristics
Source: Front Genet. 2020 Nov 30;11:565146. doi: 10.3389/fgene.2020.565146 (PMC7755106; doi:10.3389/fgene.2020.565146)
Supplement: Supplementary file 9 [file Data_Sheet_9.pdf]

Supplementary Data 9. Expression level (CPM) of 13 *CAD* family genes in leaves, flowers, stems, seedling roots, and seedling shoots of six flax cultivars/lines: #3896, Alizee, Atlant, Diplomat, LM98, and Universal. Green bars – leaves, blue – flowers, gray – stems, orange – seedling roots, red – seedling shoots.

CAD1A

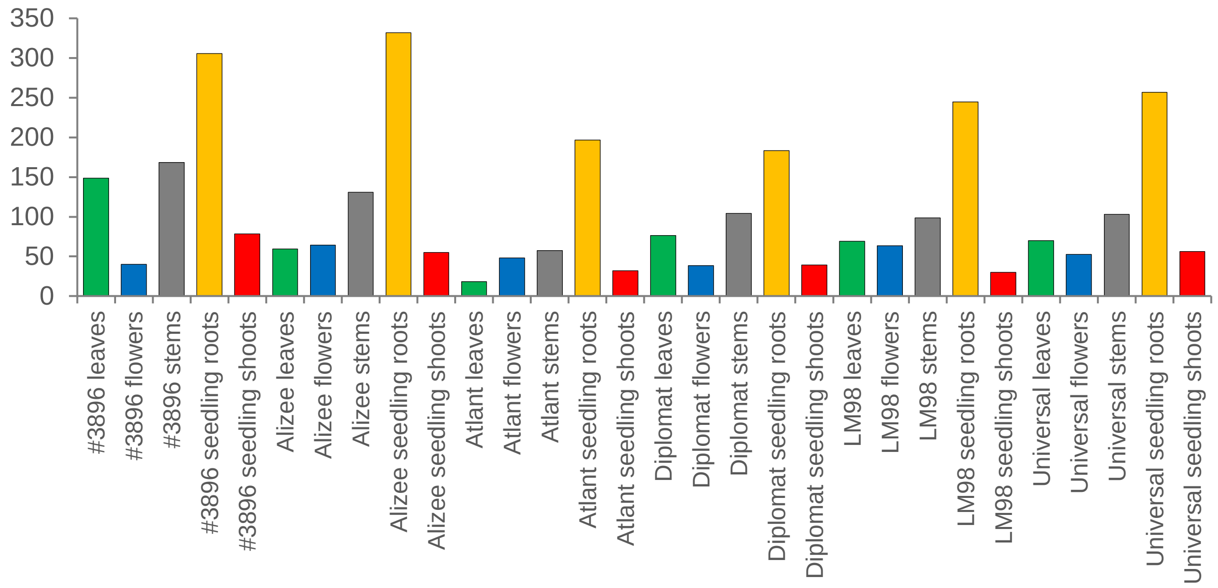

CAD1B

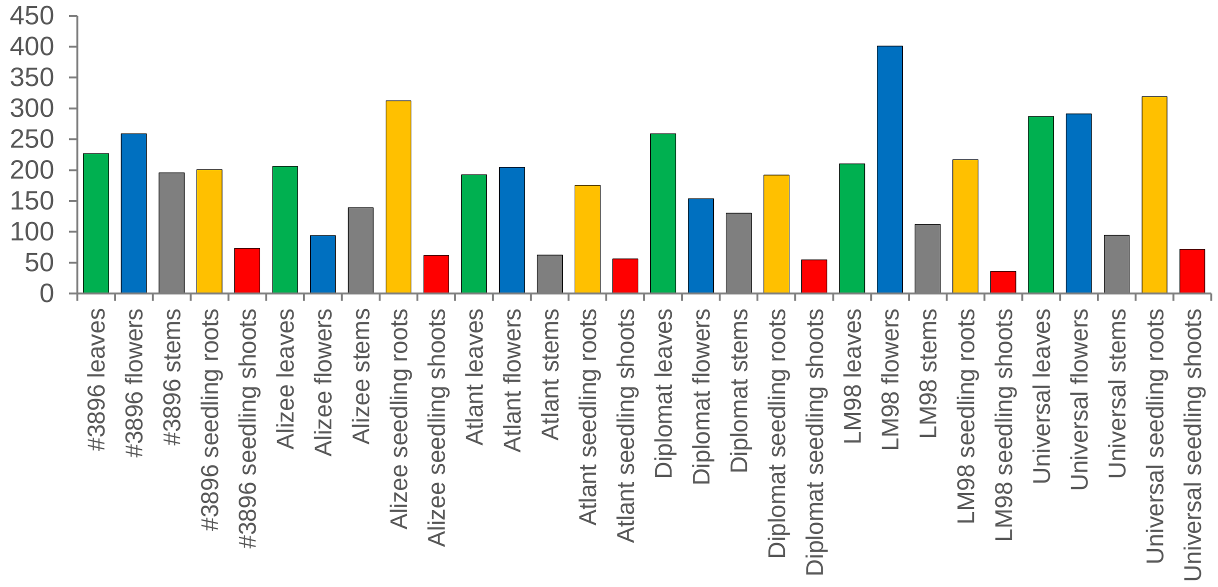

CAD2A

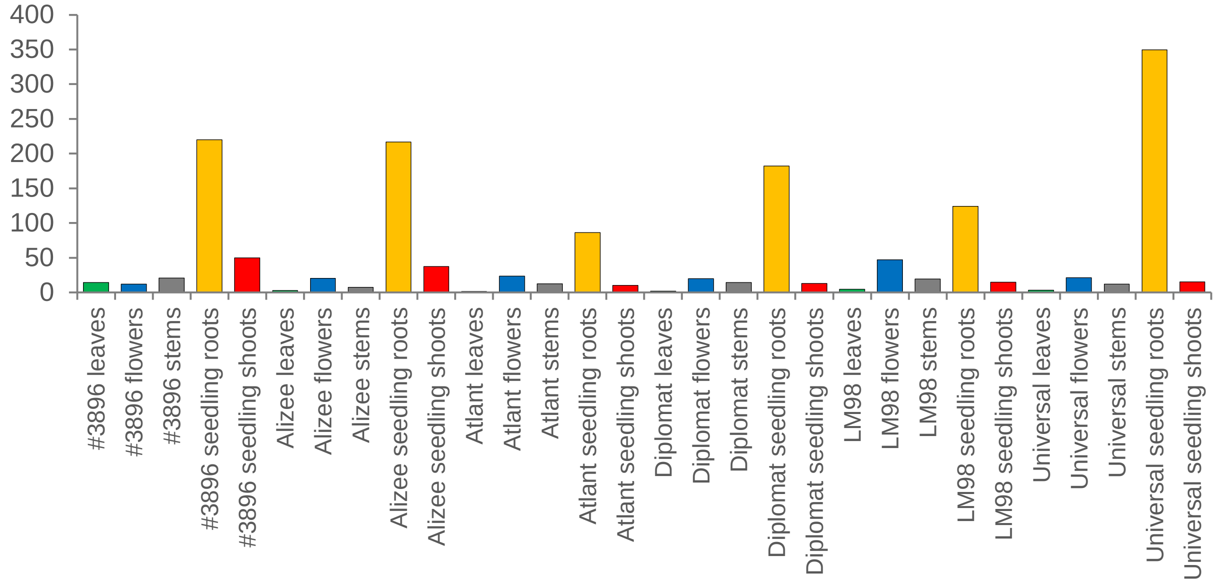

CAD2B

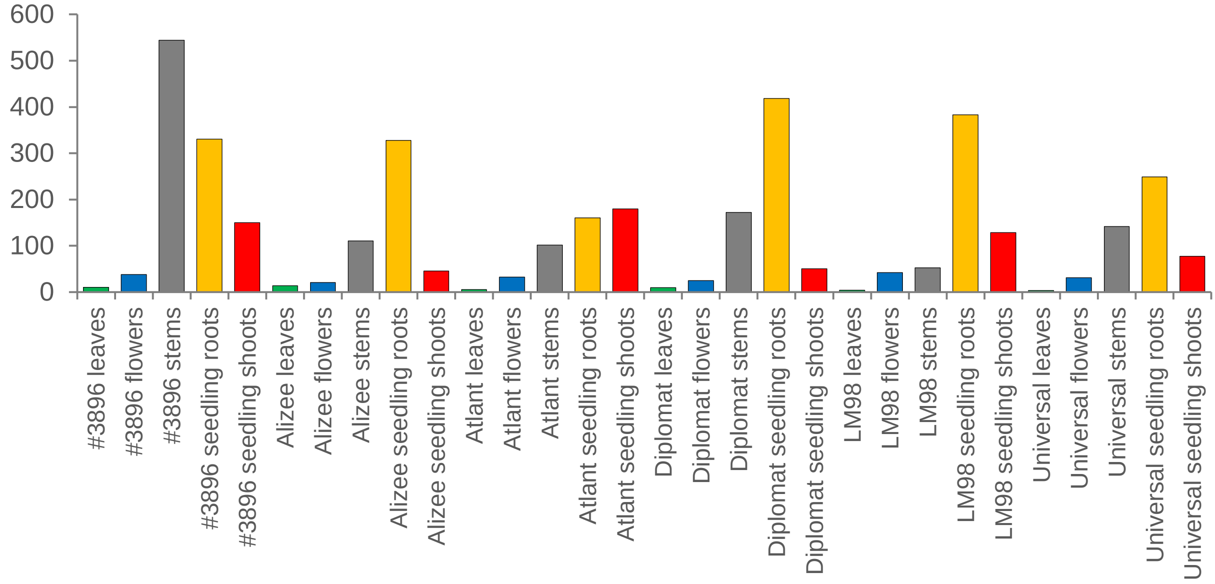

CAD3A

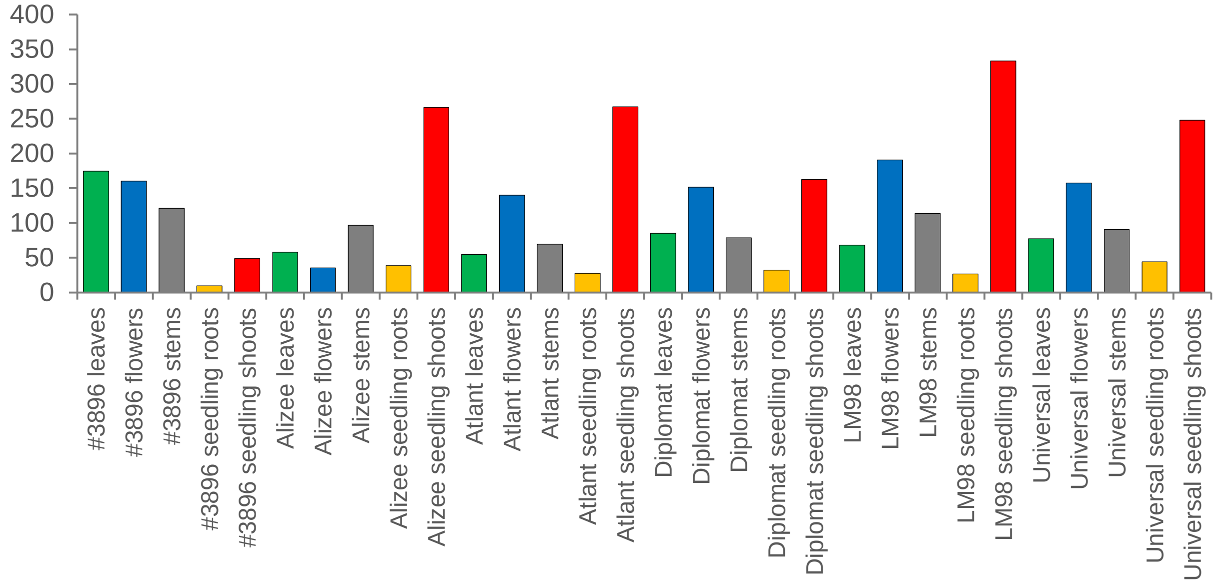

CAD3B

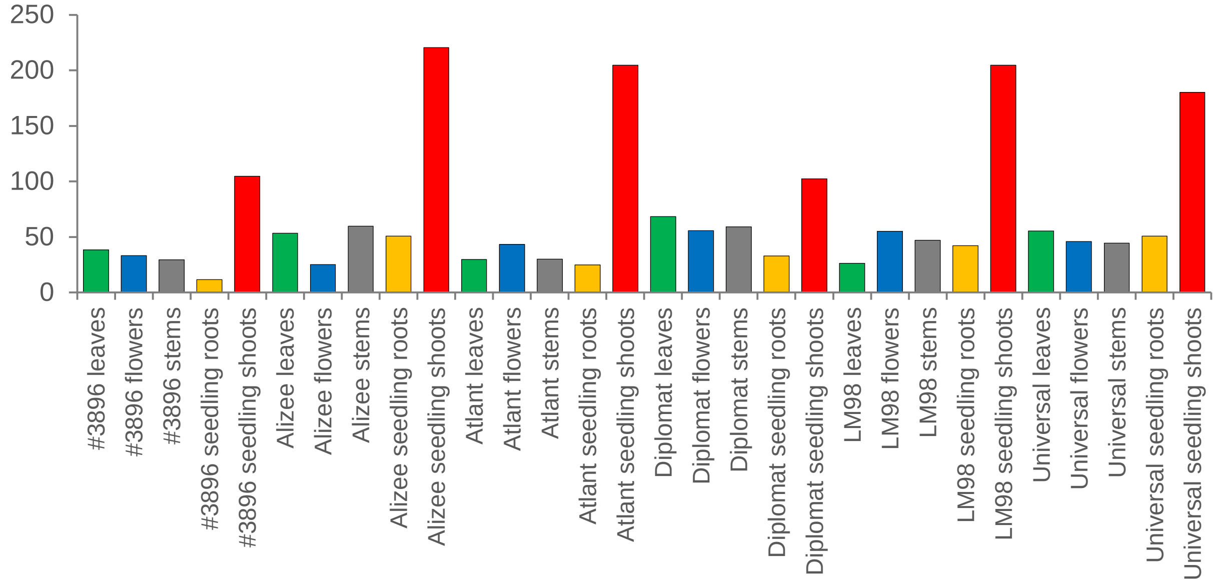

CAD4A

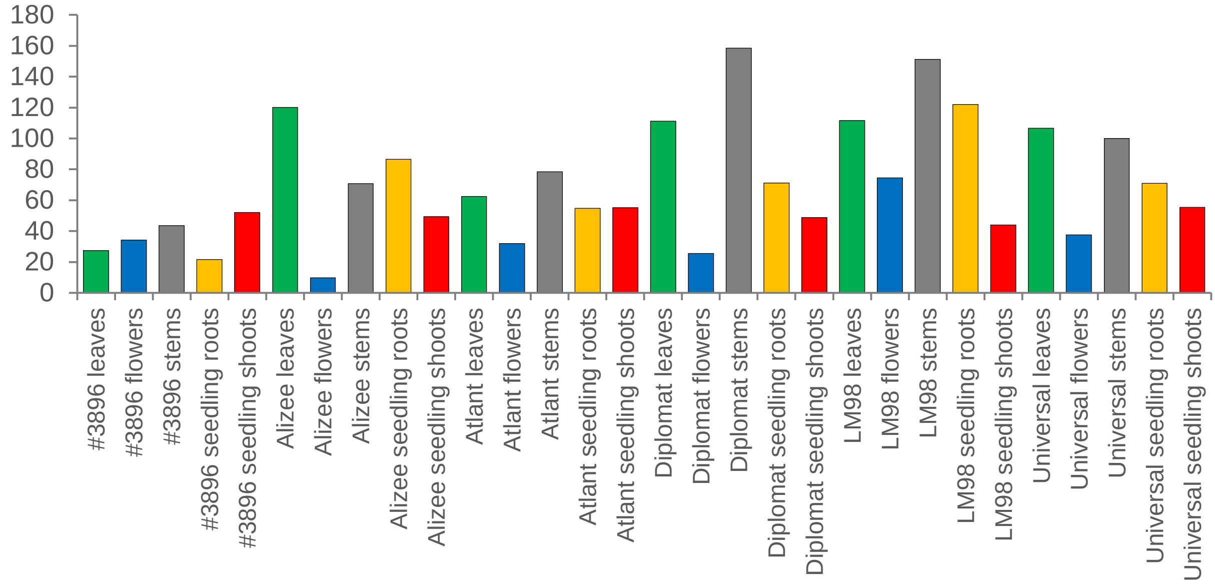

CAD4B

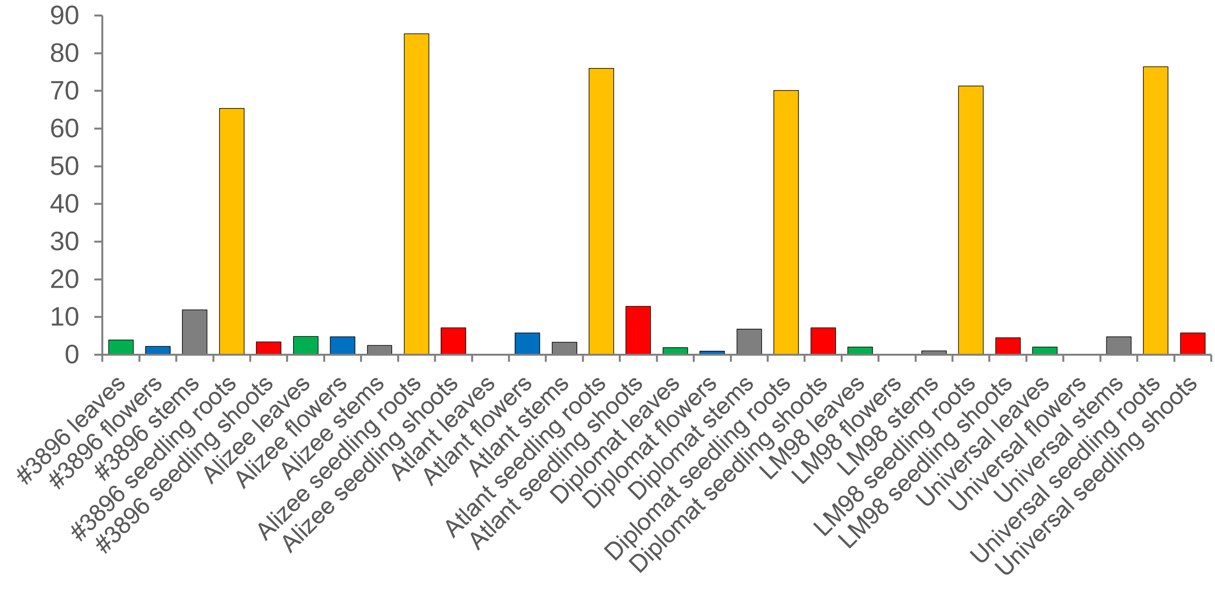

CAD5A

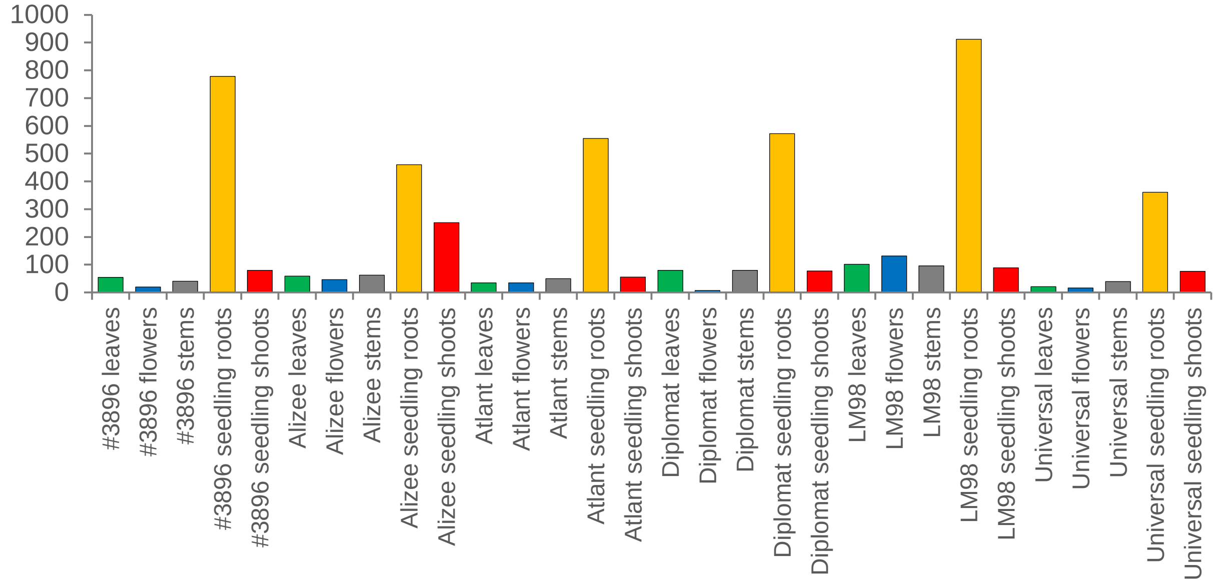

CAD5B

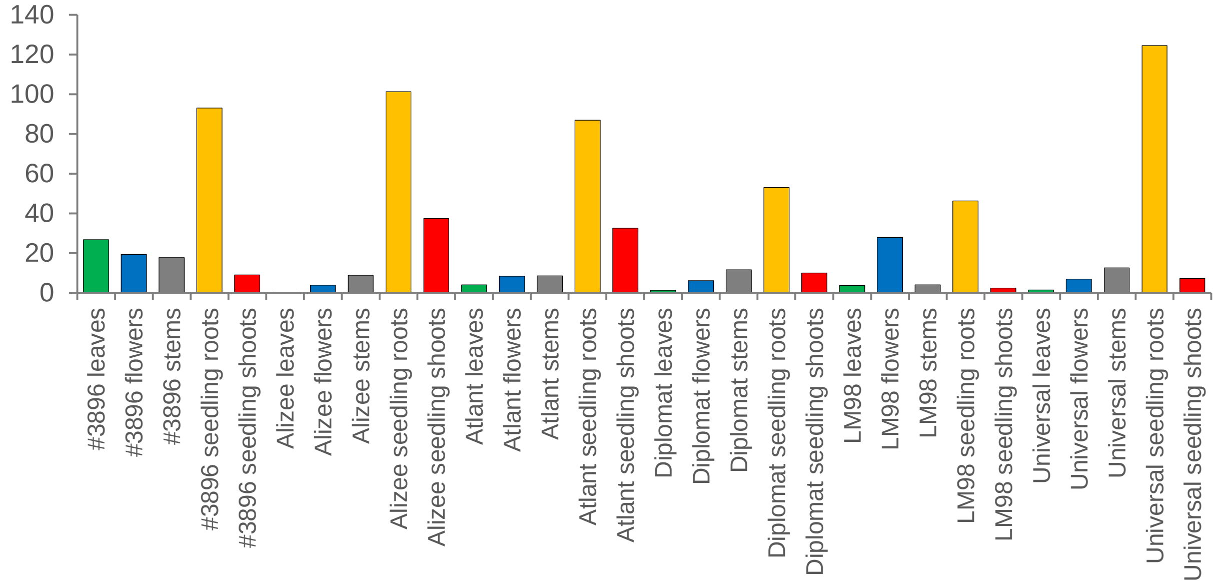

CAD6

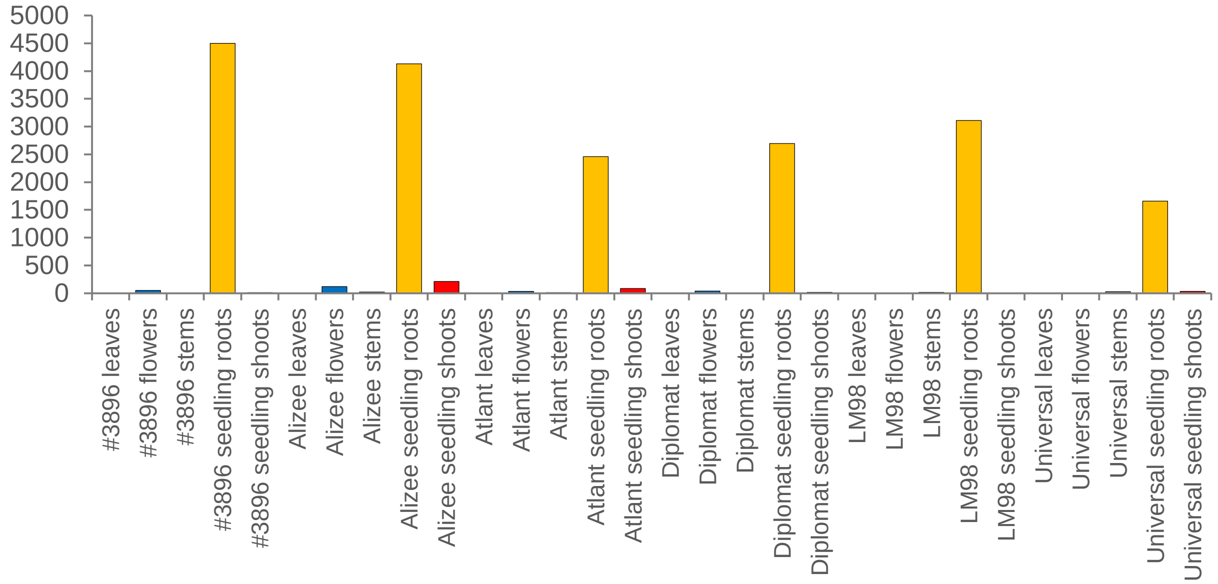

CAD7

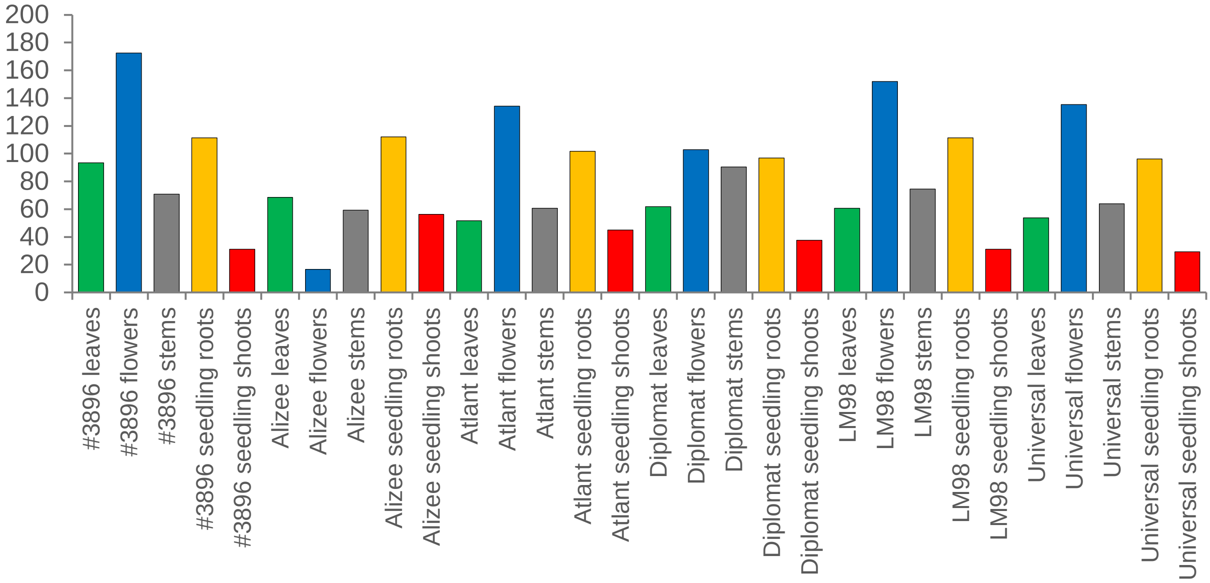

200
180
160
140
120
100
80
60
40
20
0
#3896 leaves
#3896 flowers
#3896 stems
#3896 seedling roots
#3896 seedling shoots
Alizee leaves
Alizee flowers
Alizee stems
Alizee seedling roots
Alizee seedling shoots
Atlant leaves
Atlant flowers
Atlant stems
Atlant seedling roots
Atlant seedling shoots
Diplomat leaves
Diplomat flowers
Diplomat stems
Diplomat seedling roots
Diplomat seedling shoots
LM98 leaves
LM98 flowers
LM98 stems
LM98 seedling roots
LM98 seedling shoots
Universal leaves
Universal flowers
Universal stems
Universal seedling roots
Universal seedling shoots

CAD8

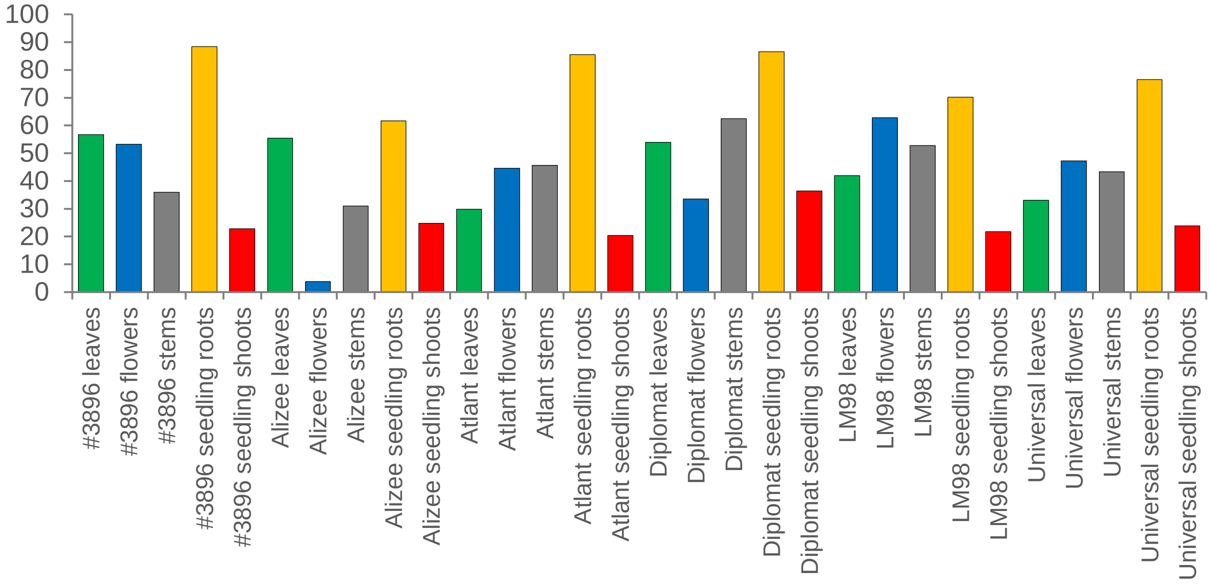

100
90
80
70
60
50
40
30
20
10
0
#3896 leaves
#3896 flowers
#3896 stems
#3896 seedling roots
#3896 seedling shoots
Alizee leaves
Alizee flowers
Alizee stems
Alizee seedling roots
Alizee seedling shoots
Atlant leaves
Atlant flowers
Atlant stems
Atlant seedling roots
Atlant seedling shoots
Diplomat leaves
Diplomat flowers
Diplomat stems
Diplomat seedling roots
Diplomat seedling shoots
LM98 leaves
LM98 flowers
LM98 stems
LM98 seedling roots
LM98 seedling shoots
Universal leaves
Universal flowers
Universal stems
Universal seedling roots
Universal seedling shoots
